# The dynamics of coastal macroalgal assemblages after the Fundão dam disaster: challenges and lessons from impact assessment

**DOI:** 10.1007/s10661-026-15191-7

**Published:** 2026-04-01

**Authors:** Ivan M. Carneiro, Guilherme M. Castro, Fernando C. Cardoso, Julia P. Curvêllo, Rodrigo T. Carvalho, Leonardo T. Salgado, Julia Kaiser, Paulo S. Salomon, Rodrigo L. Moura

**Affiliations:** 1 Instituto de Biologia and Núcleo Rogerio Valle de Produção Sustentável/COPPE, Universidade Federal Do Rio de Janeiro, Rio de Janeiro, RJ Brazil; 2 Departamento de Biologia, Pontifícia Universidade Católica Do Rio de Janeiro, Rio de Janeiro, Brazil; 3 Instituto de Pesquisas Jardim Botânico Do Rio de Janeiro, Rio de Janeiro, RJ Brazil

**Keywords:** Sampling design, Spatial scales, *Sargassum*, Brown algae, Long term ecological research

## Abstract

Anthropogenic marine disasters impair biodiversity, ecosystem services provisioning, and socioeconomic development, with long-lasting consequences. Robust impact assessment, however, depends on understanding the spatiotemporal variability of the indicators used to detect change. Here, we present the results of a monitoring program (2018–2025) targeted at two Coastal and Marine Protected Areas located 40 km south of the Doce River mouth (Eastern Brazil), which were affected by the discharge of iron ore tailings from the Fundão dam collapse in 2015. Besides assessing the dynamics of the benthic assemblages, we revealed a high variability among sampling units (residual), along with relevant variability across seasons, sites, and their interactions. Wave height and distance from rivers were the most influential drivers explaining assemblage variability. Before–after contrasts based on pre-disaster data from the literature indicate shifts in the assemblage structure. However, because baseline data are limited, the observed changes cannot be conclusively attributed to the disaster. An overview of marine areas under long-term ecological monitoring in Brazil revealed a concerning mismatch with the distribution of mining, oil and gas, port, and nuclear activities. Rethinking funding for baseline data collection and monitoring of disaster-prone marine areas, primarily from industries that are often responsible for marine disasters, constitutes a necessary step toward effective solutions.

**Supplementary Information:**

The online version contains supplementary material available at 10.1007/s10661-026-15191-7.

## Introduction

As marine pollution escalates, robust assessments of anthropogenic impacts on coastal and marine ecosystems have never been more critical (Halpern et al., 2008). Accordingly, accurate biodiversity metrics and long-term ecological monitoring are central to the Kunming-Montreal Agreement adopted at the 15th Conference of the Parties to the Convention on Biological Diversity (COP-15), which aims at informing management and funding priorities for biodiversity conservation. However, highly diverse and structurally complex marine habitats such as reefs and marine forests are still particularly challenging to assess and monitor, as they remain poorly mapped and may exhibit significant spatiotemporal variability, potentially leading to scale mismatches between the ecological processes and the resolution of available data (Bishop et al., 2002; Konno et al., 2024; Underwood, 1994; Underwood et al., 2000). These challenges become even more pronounced following anthropogenic marine disasters, where ecological consequences often display elusive trends driven by interactions among biophysical processes and multiple pollution sources across thousands of km^2^ (Franco et al., 2024; Grillo et al., 2025).

Macroalgae (seaweeds) play a pivotal role in hard bottom coastal ecosystems by modulating biodiversity and participating in nutrient and carbon cycling (Hurd et al., 2014; Ólafsson, 2017). They are also well-established indicators of eutrophication and contamination by heavy metals and other trace elements (*e.g.*, D’Archino & Piazzi, 2021). Macroalgae assemblages often exhibit considerable seasonal fluctuations (Hurd et al., 2014; Ólafsson, 2017), particularly in the intertidal and upper-subtidal zones dominated by fast-growing filamentous forms or pseudo-perennial canopy-forming algae like *Sargassum*, which shed lateral branches after reproductive peaks or stormy seasons (Széchy et al., 2006; Thomsen et al., 2006; Titlyanov et al., 2014). Sedimentation, seawater temperature, wave action, and proximity to rivers or urbanized areas, along with coastal pollution, are among the most influential drivers of spatiotemporal variability in these assemblages (Hurd et al., 2014; Pardal et al., 2023). While seawater temperature operates across larger spatial scales (*e.g.*, Cordeiro et al., 2024), wave action operates more locally by dislodging perennials and favoring opportunists, constraining morphology and size through drag forces, and modulating interactions with sediments (Hurd et al., 2014; Burel et al., 2019).

In Brazil, benthic assemblages have been more comprehensively assessed in the rocky shores that dominate the shoreline between Santa Catarina and Cabo Frio (*e.g.,* Carneiro et al., 2021; Pardal et al., 2023), but information on seasonal variation remains restricted to a few sites in the intertidal and shallow-subtidal zones (e.g., Guimaraens & Coutinho, 1996; Amado-Filho et al., 2003, 2008) and one longer-term (7-year) study in an estuarine intertidal setting dominated by *Ulva* spp. (Puga et al., 2019). Northward, across the subtropical-tropical transition along the Espírito Santo State coast, most published data stem from snapshot assessments (e.g. Mazzuco et al., 2020; Scherner et al., 2013; Vassoler et al., 2025). Nearshore reefs along the Espírito Santo shoreline are formed by laterites intermingled with coralline algae and coral pavements and host the highest macroalgal biodiversity in the Western South Atlantic, with over 500 recorded taxa (Karez et al., 2023).

The Fundão dam collapse (November 5, 2015) stands as one of the world’s worst mining disasters, releasing approximately 40 × 10^6^ m^3^ of iron ore tailings into the Doce River watershed and severely impacting the livelihoods of up to 3 million people through displacement in the most directly impacted communities and widespread disruption of drinking-water supplies and river-dependent activities, especially fishing and agriculture (Franco et al., 2024; Freitas et al., 2022). While nearly half of the tailings were held back at an upstream hydroelectric dam, a massive slurry moved 650 km downstream toward the estuary, leading to increased concentrations of trace elements in water, sediments and marine organisms (Gabriel et al., 2020; Lopes et al., 2025). Impacts have been documented across the watershed, estuary, beaches and marine soft sediments (Andrades et al., 2020; Brahim et al., 2024; Nascimento et al., 2022; Richard et al., 2020), and contamination has been demonstrated in invertebrates, marine fishes, turtles, birds and mammals (Bevitório et al., 2022; Cardoso et al., 2022; Lopes et al., 2025; Manhães et al., 2022; Miguel et al., 2022; Nunes et al., 2022). Anomalies in iron and other trace elements’ concentrations were recorded after 2015 in corals collected 220 km northwards of the river mouth (Cardoso et al., 2022). Franco et al. (2024) provided a thorough *ex post* evaluation of the disaster’s ecological consequences, highlighting the challenges of assessing impacts and recovery, emphasizing the importance of establishing adaptive monitoring programs that could be updated based on evolving environmental conditions. This aligns with the insights of Martinez et al. (2022a, 2022b) and Choueri et al. (2022), who discussed how the absence of control sites and proper replication in impact assessments complicates the interpretation of long-term ecological consequences. The lack of baseline studies on coastal and marine ecosystems that received contaminants from the Fundão Dam collapse led to a decade-long and yet unsettled debate over the extent of contamination and its ecological and socioeconomic impacts (Cardoso et al., 2022; Franco et al., 2024; Primo et al., 2021), with up to 250 million USD already allocated for community support and long-term conservation initiatives.

Capturing ecological patterns and scale-dependencies is crucial to enhance the understanding of ecosystem dynamics and improve monitoring, mitigation, and restoration strategies in the aftermath of the Fundão disaster. This study aims to assess the spatiotemporal variability of benthic assemblages across spatial scales ranging from meters (within-site variability) to tens of kilometers (among sites) following the dam collapse, and to examine potential drivers of this variability and its impacts. The implications of limited baseline data were discussed in the context of the assessment of the disaster impacts and Brazil’s environmental monitoring policies. Spatiotemporal variability was accessed using a hierarchical sampling design in which seasons were nested within years and crossed with sites. Since proximity to rivers, seawater temperature, turbidity, and wave exposure are known drivers of variability in benthic assemblages (Hurd et al., 2014; Pardal et al., 2023), we hypothesized that spatial differences among sites would reflect underlying environmental gradients associated with these factors. Our results were compared with a snapshot of pre-disaster benthic cover from the only two sites where baseline data were available (Scherner et al., 2013). The mismatches between disaster-prone areas and long-term monitoring efforts were assessed, highlighting significant gaps in Brazil’s environmental monitoring policies under increasing urbanization and industrialization.

## Material and methods

### Study area

**Fig. 1:**
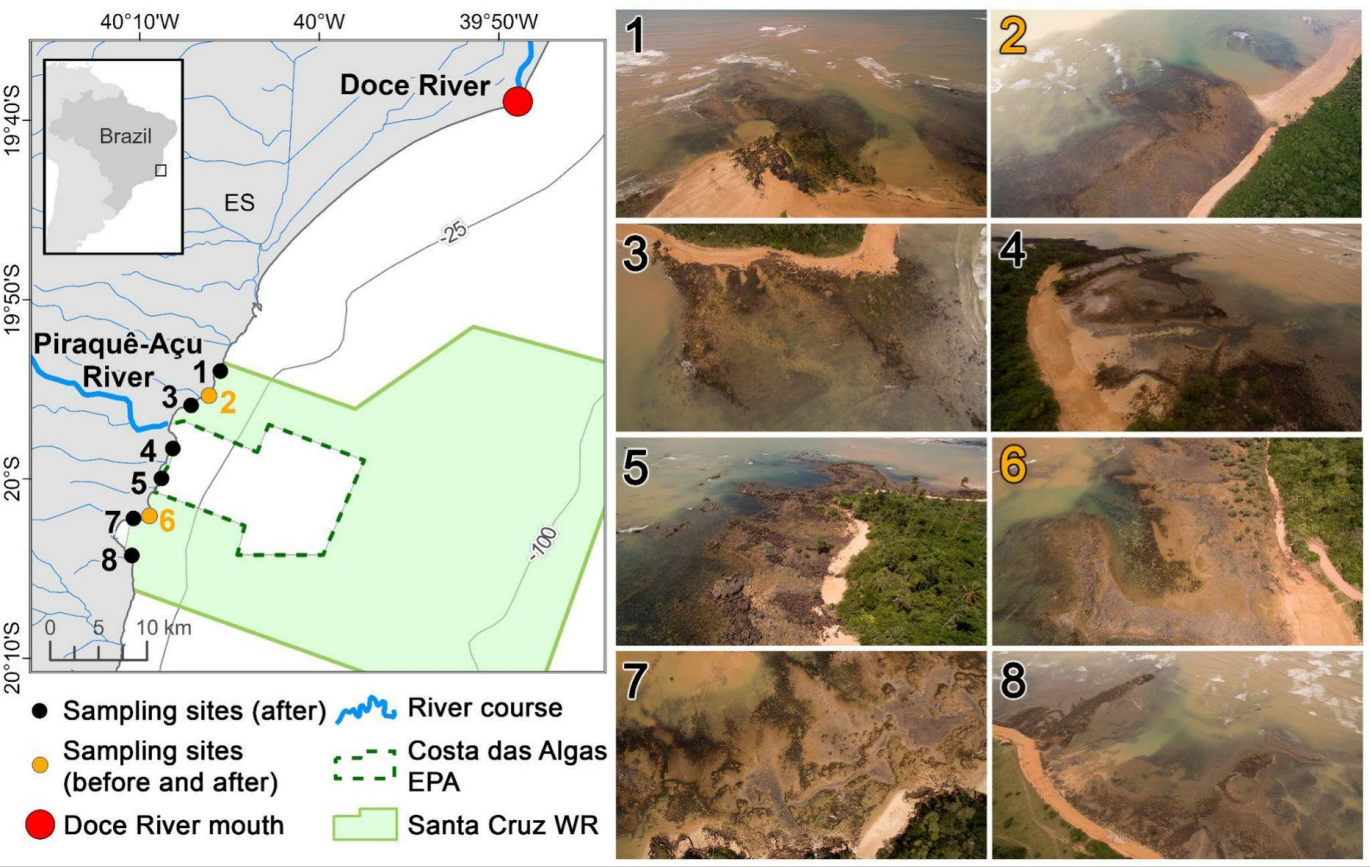
Location and characteristics of the reef sites sampled between 2018 and 2025. Sites sampled before and after the disaster are highlighted (orange) and the Doce River mouth is indicated with a circle (red). Isobaths (25 and 100 m) and Protected Areas are also shown. Numbers in aerial photographs (1–8) correspond to those in the map. Photos by the authors

The study region comprises a 30 km coastal stretch in Southeast Brazil, dominated by beaches and fringing reefs, located 40 km south of the Doce River mouth and 10 km south of a fishing-ban zone established in 2016 due to human health risks associated with the dam collapse (Fig. 1). The eight studied sites are located within two contiguous Coastal and Marine Protected Areas (CMPAs), the multiple-use Costa das Algas (*Algae Coast*, in English) Environmental Protection Area and no-take Santa Cruz Wildlife Refuge.

Lateritic rocks, sandstones, and limestones comprise most of the physical structure of the reefs, which are under a 0.9 to 2.2 m tidal range (neap and spring tide, respectively) and strong continental and riverine influence. The geomorphology of these reefs contrasts sharply with Southeastern Brazil’s rocky shores, which are dominated by boulders, cliffs, and abrasion terraces formed by crystalline rocks (Coutinho & Christofoletti, 2024). Sampling sites encompass intertidal reef flats with hundreds of meters width and numerous shallow tidepools and channels (<1 m depth).

The Doce River is the main freshwater and sediment source, with an annual average discharge of 850 m^3^ s^−1^ (Quaresma et al., 2020). Two smaller rivers, Piraquê-Açu and Reis Magos, also discharge near the studied reefs. Southerly waves predominate between April and September, when wave heights and periods are greater (Vinzon et al., 2023). Northerly winds predominate across the year, but tidal currents and hydraulic jetty effects facilitate longshore northward transport, particularly during S-SW cold fronts’ intrusions (Albino & Suguio, 2010).

### Sampling

Coastal and marine monitoring began 3 years after the dam rupture (Franco et al., 2024), precluding the detection of short-term responses. Between September 2018 and March 2025, we conducted two annual surveys (first and second semesters, respectively) covering the eight sites (Fig. 1). Fieldwork was not carried out in 2020 and the second semester of 2021 due to COVID restrictions and logistic constraints. Benthic cover was estimated using 0.5 m^2^ photoquadrats with 15 contiguous images taken with a Canon G-15 camera (macro mode, highest resolution) and two strobes. Photoquadrats were randomly distributed during low tides at each site, from the lower intertidal to the upper subtidal. Once the studied reefs are not a typical rocky shore with an overall vertical physical structure (Coutinho & Christofoletti, 2024), we applied a random sampling design covering the intertidal domain of the reef flat and its shallow channels and tidepools, down to approximately 1 m below the lowest astronomical tide.

Images were visually annotated in the CoralNet platform (Beijbom et al., 2015) using 225 randomly distributed points per photoquadrat. Benthic cover was quantified as percent cover of each benthic category. Macroalgae were categorized into seven functional groups following Steneck and Dethier (1994): articulated calcareous, crustose, corticated, corticated foliose, leathery, filamentous, and foliose. Sessile macroinvertebrates were merged as a single response variable rather than classified into separate taxonomic or functional categories. In total, 12 benthic categories were considered, including the seven macroalgae functional groups, turf, seagrass, sessile macroinvertebrates, sediment, and unknown macroalgae. Two complementary approaches were used to analyze the data, one to assess spatiotemporal variability across multiple spatial scales after the disaster, and the other to perform before–after contrasts using the limited pre-disaster data (SM Table 1).

### Spatiotemporal variability and environmental drivers

Benthic cover of all sites and semesters was compared using multivariate permutational analyses of variance (PERMANOVA), with semesters nested within years (*n* = 4 years and 8 sites with data from both semesters) (SM Tab. 1A). PERMANOVAs were run in software PRIMER 7 + PERMANOVA (Anderson et al., 2008), based on Bray–Curtis distances and 999 random permutations. In order to assess variability across spatiotemporal scales, variance component (Anderson et al., 2008) was calculated for each factor (site, year and semester nested within year) and interactions. The residual variability represents the variation among sampling units located 3–20 m apart within each site (5 to 15 sampling units per site/semester). Data for each site, year and season are available in a Shiny application for visualization and download (Chang et al., 2025; Carneiro et al., 2026). Proximity to rivers (Doce, Piraquê-Açu, and Reis Magos), average surface seawater temperature (SST), turbidity, and wave heights in the 3 months preceding each survey were evaluated as possible drivers of variability (*n* = 4 years and 8 sites with data from both semesters). Turbidity (Diffuse Attenuation Coefficient, 490 nm) and SST were extracted from the MODIS and S-NPP/VIIRS sensors, respectively (9 km resolution). Average wave height was extracted from the ERA5 re-analysis of the global climate (Hersbach et al., 2020) and corresponds to the average of hourly significant wave heights for each 3-month period. Distance from Reis Magos River mouth was excluded due to its high correlation with that of the Doce River. A Distance-Based Redundancy Analysis (db-RDA) preceded by a stepwise selection of significant predictors was employed to explore the relationship between environmental variables and benthic cover, using the *vegan* package in the R environment (Oksanen et al., 2019; R Core Team, 2023).

### Macroalgae assemblages before and after the disaster

Macroalgal assemblages were contrasted considering data obtained by Scherner et al. (2013) in sites 2 and 6 in the summer of 2012, 3 years before the disaster (SM Tab. 1B). Their samples consisted of three replicated photo-transects (30 photographs with 25 × 25 cm along a line) at each site, totaling 5.62 m^2^, and were compared with our summer data from the same two sites. Our data includes samples acquired in the summers of 2019, 2021, 2023, and 2025 (3, 5, 7, and 9 years after the disaster, respectively). Samples from April 2022 and 2024 were disregarded, as they were obtained in autumn. In 2019 and 2021, five photoquadrats were randomly distributed at each site (2.5 m^2^), while 15 (7.5 m^2^) and 10 (5 m^2^) photoquadrats were sampled in each site in 2023 and 2025, respectively. To ensure consistency in the before–after contrasts, we used the identifications reported by Scherner et al. (2013) and reclassified them into our functional categories. Sediment cover was not reported by Scherner et al. (2013) and was excluded from the comparisons.

Pre- and post-disaster benthic cover samples at sites 2 and 6 were compared using PERMANOVA and pairwise post-hoc tests. Benthic cover was contrasted with Principal Coordinate Analyses (PCoA) using the software PRIMER 7 + PERMANOVA (Anderson et al., 2008).

### Disaster risk and long-term monitoring

Since 1998, Brazil has established coastal and marine long-term ecological research (LTER) “sites” funded by the Brazilian National Research Council (*Conselho Nacional de Desenvolvimento Científico e Tecnológico,* CNPq) and state agencies, the so-called Long-Term Ecological Research Program (PELD). Spatialization of LTER sites and operations that may cause disasters was used to evaluate trends and mismatches. Operations included mining dams, oil wells and fields/blocks, ports (operating and under construction), and nuclear powerplants, all of which are responsible for marine disasters around the globe (sources and filters in SM Tab. 2). Distances among LTER sites were calculated to quantify spatial gaps in long-term monitoring. Distances between each LTER site and the nearer mining dams, nuclear powerplant, oil wells and ports under operation were calculated, tallying the number of operations related with each site. For mining dams, distances were measured from the mouth of the river draining each dam to the nearest LTER site.

## Results

### Spatiotemporal variability and environmental drivers

During the sampling period, reef cover was dominated by macroalgae, especially leathery (i.e., *Sargassum* spp*.*), foliose corticated (i.e., *Padina* spp*.* and *Dictyota* spp*.*), foliose (*Ulva* spp.), and articulated calcareous (i.e., *Amphiroa* spp*.* and *Jania* spp*.*) forms. Unknown macroalgae represented less than 1% of benthic cover at each site and were not relevant contributors to spatiotemporal variability. Sessile invertebrates, mostly zoanthids (*Palythoa caribaeorum* and *Zoanthus* spp.) and sparse coral colonies (primarily *Siderastrea* sp.), generally exhibited less than 5% cover in most sites. The hierarchical PERMANOVA revealed significant differences between semesters, sites, as well as an interaction between semesters and sites (Table 1). Variance components highlighted the highest residual variability (58%), followed by the interaction between semesters and sites (24%), among sites (9%), and between semesters within the same given year (8%). Conversely, there was low variability among years (1%) and negligible variability associated with the interaction between site and year (Table 1).

**Table 1:** Results of the hierarchical PERMANOVA comparing benthic cover considering factors site (Si), year (Yr) and semester (Se). Significant differences (*p* < 0.05) are in bold. Df = degrees of freedom; MS = mean square; P(perm) = *p*-value estimated by permutation; VC = variance component

Source	df	MS	Pseudo-F	P(perm)	Unique permutations	VC (%)
Si	7	16,221	3.03	**0.001**	998	9
Yr	3	11,754	1.20	0.365	844	1
Se(Yr)	4	10,086	9.45	**0.001**	999	8
Si x Yr	21	5464	0.99	0.479	997	0
Si × Se(Yr)	23	5069	4.75	**0.001**	997	24
Residual	570	1067				58
Total	628					

**Fig. 2:**
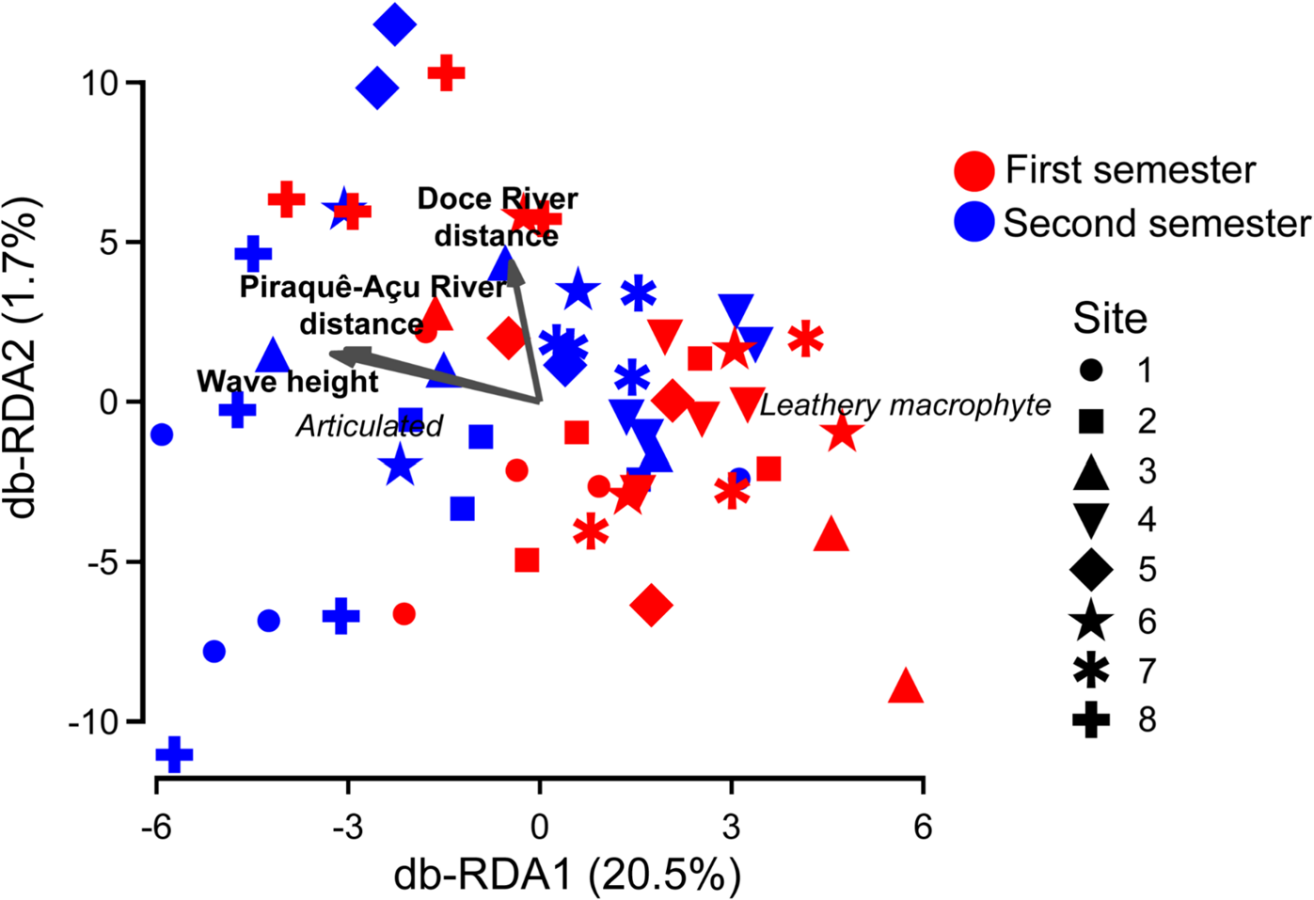
Distance-based redundancy analysis (db-RDA) indicating the relationship between the stepwise-selected environmental variables and assemblage structure. Most important macroalgae functional groups (r^2^ > 0.80) are highlighted

The stepwise selection of variables revealed that benthic cover was significantly related to the distance from the mouth of rivers Doce and Piraquê-Açu, and average wave height (Table 2). The db-RDA evidenced that these three variables explained 22.2% of the total variability (20.5 and 1.7% for the first and second axes, respectively) (Fig. 2). Furthermore, leathery macroalgae were closely aligned with samples from the first semesters, when wave heights are lower, while articulated coralline algae were associated with higher wave heights (Fig. 2).

**Table 2:** Environmental variables selected by the stepwise procedure

	df	AIC	F	*p*-value
Distance from Doce River mouth	1	382.23	3.85	**0.010**
Average wave height	1	385.03	6.71	**0.005**
Distance from Piraquê-Açu River mouth	1	386.85	8.64	**0.005**

### Macroalgae assemblages before and after the disaster

**Fig. 3:**
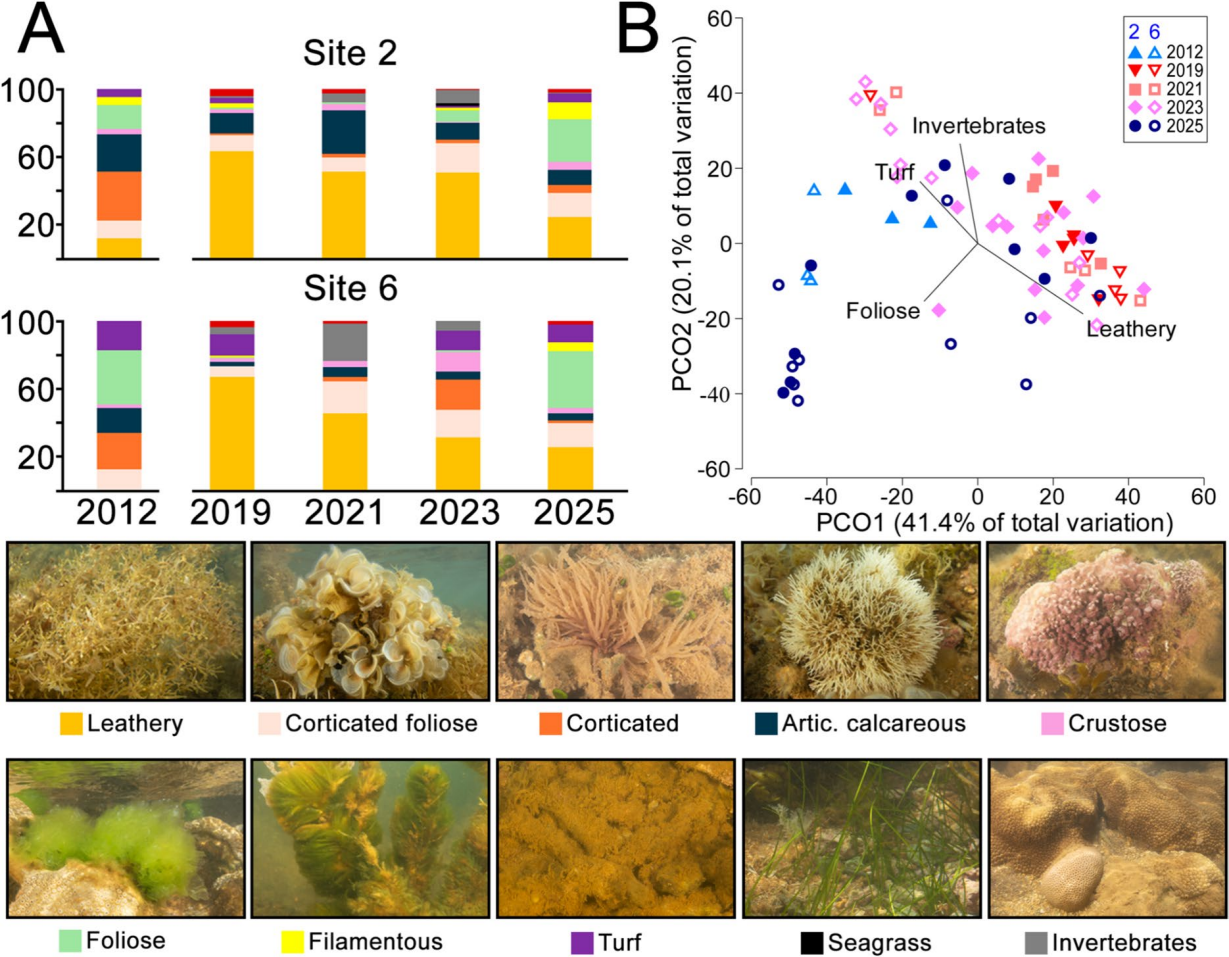
Temporal dynamics of benthic assemblages at the two sites with pre-disaster baselines (2 and 6). **A** Percent cover of macroalgae functional groups and invertebrates. **B** Principal Component Analysis (PCoA) contrasting sampling units from before (2012) and after (2019–2025) disaster. The lower panel shows representative organisms of the seven macroalgal functional groups, turf, seagrass, and the two most abundant invertebrates (*Palythoa caribaeorum*—dominant in the image and *Siderastrea* sp.—lower left). Colored legends of groups correspond to the stacked bars in the upper left panel (**A**). Cover of unknown macroalgae indicated by red bars (**A**)

Considering summer data from the two sites with baselines (2 and 6), we observed significant variation both between years and sites (Fig. 3; Table 3). Pairwise comparisons revealed differences between the 2012 baseline and all years after the disaster, except for 2025 (Table 4). No interaction was found between sites and years. Leathery macroalgae comprised the most abundant functional group after the disaster and are associated with the first and second PCoA axis, which explained approximately 40% and 20% of the variation, respectively. Leathery macroalgae abundance declined at both sites since 2019, especially at site 6 (Fig. 3A).

**Table 3:** Summary of the PERMANOVA comparing benthic cover before (2012) and after (2019, 2021, 2023 and 2025) the disaster in the two sites with baselines (2 and 6). Df = degrees of freedom; SS = sum of squares; MS = mean square; P(perm) = *p*-value estimated by permutation. Significant differences (*p* < 0.05) in bold

	Df	SS	MS	Pseudo-F	P(perm)
Site (Si)	1	7419	7419	5.44	**0.002**
Year (Yr)	3	20,727	6909	5.07	**0.001**
Si × Ye	3	4705	1568	1.15	0.304
Residuals	55	75,011	1364		
Total	62	107,860

**Table 4:** Pairwise contrasts (PERMANOVA) between years in sites 2 and 6, sampled during summers before (2012) and after (2019, 2021, 2023 and 2025) the disaster. P(perm) = *p*-value estimated by permutation. Significant differences (*p* < 0.05) in bold

	Site 2		Site 6	
	*t*	P(perm)	*t*	P(perm)
2012, 2019	3.6894	**0.02**	2.7203	**0.013**
2012, 2021	2.8136	**0.012**	2.0255	**0.048**
2012, 2023	2.7573	**0.002**	1.7669	**0.011**
2012, 2025	1.2453	0.202	1.509	0.072
2019, 2021	1.6453	0.082	1.1047	0.19
2019, 2023	1.3816	0.107	1.509	0.063
2019, 2025	1.938	**0.028**	2.2502	**0.012**
2021, 2023	1.3734	0.124	1.0978	0.32
2021, 2025	2.0562	**0.012**	2.0105	**0.016**
2023, 2025	2.369	**0.006**	2.4293	**0.001**

### Disaster risk and long-term monitoring areas

**Fig. 4:**
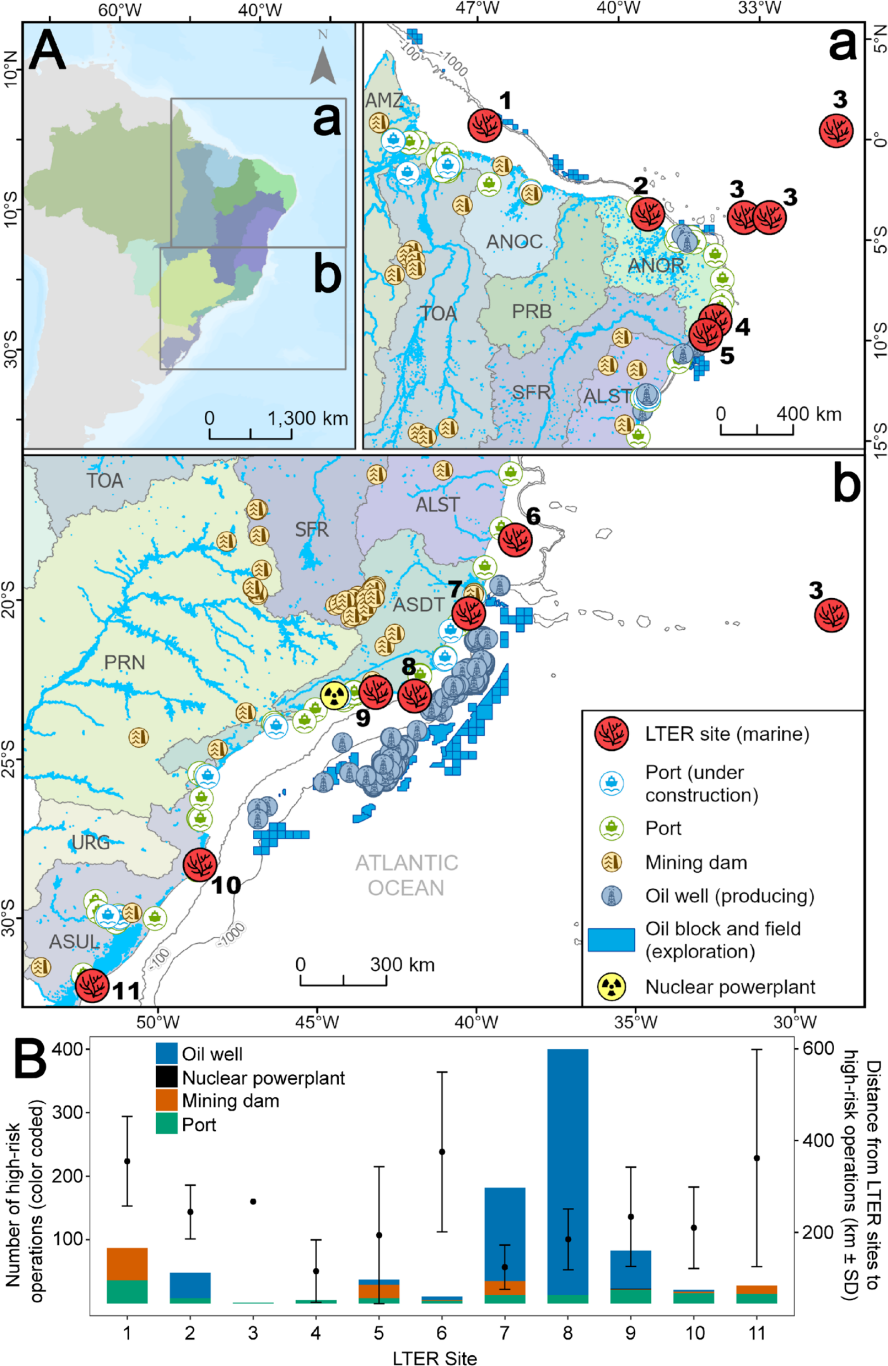
**A** Brazilian marine areas with long-term ecological research (LTER/PELD) sites and operations that may cause disasters (ports, mining dams, oil and gas, and nuclear power plants). **B** Number of risky operations and mean distance to the nearest LTER site. LTER/PELD sites: 1) GARS, Sistema de Recifes Mesofóticos da Foz Rio Amazonas; 2) CSA, Costa Semi-Árida; 3) ILOC, Ilhas Oceânicas; 4) TAMS, Tamandaré Sustentável; 5) CCAL, Costa dos Corais Alagoas; 6) ABRS, Abrolhos; 7) HCES, Habitats Costeiros do Espírito Santo; 8) RLAC, Ressurgência de Arraial do Cabo; 9) BG, Baía de Guanabara; 10) SELA, Sistema Estuarino de Laguna; 11) ELPA, Estuário da Lagoa dos Patos (source: http://peldcom.eco.br)

Three of the four industrial activities responsible for major marine disasters around the world (Oil & Gas, Ports, Mining) are widespread in Brazil, while a nuclear powerplant is only present on the country’s Southeast coast (Fig. 4).

In Brazil’s Northern and Northeastern regions (Fig. 4A, insert a), disaster risk is primarily associated with ports, mining, and oil blocks. There are significant gaps (~1000 km of coastline) in the coverage of LTER sites. Mean distance from operations that may cause disasters to the nearest LTER site in these two regions ranged between 116 and 350 km (Fig. 4B). The highest proximity from port and mining operations was associated with LTER site 1, whereas proximity to operating oil wells was greatest at LTER site 2. Oceanic islands are well covered by LTER sites and are overall distant from the operations considered herein. The East, Southeast, and South Brazilian coasts (Fig. 4A, insert b) concentrate most ports and oil activities and encompass one nuclear power plant. Despite the presence of six LTER sites, there exists a substantial (900 km) monitoring gap between São Paulo and Paraná states. Mean distance to high-risk operations ranged between 125 and 375 km (Fig. 4B). With the exception of oceanic islands (site 3), all LTER sites had nearby ports. Proximity to oil wells is greater for LTER sites 7, 8, and 9, along the coasts of Rio de Janeiro and Espírito Santo, whereas mining-dam risks were primarily associated with LTER site 7, near the Doce River mouth.

## Discussion

Establishing a robust design to assess the impacts of the Fundão dam collapse on macroalgal assemblages in nearshore fringing reefs is strongly constrained by the limited availability of baseline data spanning multiple spatial and temporal scales (Underwood, 1994; Underwood et al., 2000). Nevertheless, the present study provides a detailed assessment of the post-disaster spatiotemporal variability in these assemblages and incorporates before–after comparisons where baseline information was available, enabling inferences about potential drivers and impacts in a Southwestern Atlantic hotspot of macroalgal diversity. The high variability observed between sampling units separated by only a few meters, as reflected in the residual variance component, is a well-documented pattern in reef assemblages (Fraschetti et al., 2005; Carneiro et al., 2021, 2024a). Such small-scale heterogeneity is often attributed to a myriad of interspecific interactions, variability in zygote settlement, and substrate complexity, among other potential drivers (Benedetti-Cecchi, 2001; Coleman, 2002). For instance, zygotes of *Sargassum* usually settle close to fertile adults, resulting in distribution patches that enhance small-scale spatial variability (Carneiro et al., 2020). The architecture of the studied reefs, with microhabitats with varying light availability, sedimentation and wave influence (Mazzuco et al., 2020), also contributes to the high small-scale variability, which may be accentuated by the diffuse boundary between the lower intertidal and upper subtidal in large tidal pools (Lubchenco, 1980; Murray et al., 2006).

Variation between semesters and among sites captures additional spatiotemporal scales relevant to the questions addressed here (Hartnoll & Hawkins, 1980). Despite the known influence of temperature on macroalgae phenology (Hurd et al., 2014; Széchy et al., 2006), seasonal variability in macroalgal community structure was mostly driven by increased wave heights during winter, confirming the major role of hydrodynamics (Cordeiro et al., 2024; Pardal et al., 2023). Besides dislodging and damaging sessile organisms, wave-induced disturbance affects both top-down (e.g. grazing) and bottom-up controls (e.g., sedimentation) (Jonsson et al., 2006; Lubchenco, 1980). The variation between sites seems associated with the distance from rivers Doce, which exerts regional-level influence, and Piraquê-Acu, which interferes in its vicinities. Rivers are the major conduits of land-based contaminants toward the ocean (Gabriel et al., 2020) and modify salinity, temperature, turbidity, and nutrient inputs, thereby influencing macroalgae diversity and growth (Borburema et al., 2020; Fraschetti et al., 2005; Lana & Bernardino, 2018).

Because the strong seasonality was quantified only after the disaster, differences between control and impact locations or between pre- and post-impact periods can be easily confounded (Bishop et al., 2002; Chapman et al., 1995). For example, if a before-after sampling design is employed with pre-impact samples taken during summer and post-impact samples taken in different seasons, seasonal variations may be incorrectly attributed to the impact. Conversely, impacts can remain undetected if post-disaster samples encompass pronounced variability. This latter scenario is a typical Type II error that violates the precautionary principle in environmental impact assessment.

The high abundance of leathery macroalgae in the first years after the disaster likely resulted from multiple interacting mechanisms and indirect effects, contrasting with the long-term decline of these algae along the tropical and subtropical Brazilian coast, which has been linked to climate change, urbanization, and thermal pollution (Carneiro et al., 2024b; Gorman et al., 2020). Although BACI studies of macroalgal assemblages under acute impacts from mining are lacking, observational studies evidenced that brown algae present an overall high tolerance to heavy metals. For example, *Sargassum* tolerates chronically elevated Cd and Zn in Southeastern Brazil (Amado-Filho et al., 1997, 1999), and populations of the brown alga *Lessonia* remained stable under exposure to iron-rich tailings in Chile (Vásquez et al., 1999). On the other hand, grazing is a major driver of brown algae abundance in intertidal habitats with tidal pools and upper subtidal zones (Lubchenco, 1980). In our study, disruptions to grazer populations due to contaminant toxicity (Lopes et al., 2025), together with sedimentation from the disaster (Quaresma et al., 2020; Richard et al., 2020), may have contributed to leathery macroalgae dominance. Sediments function as a reservoir of contaminants, and grazing sea urchins have shown persistent and significant toxicological effects following the dam rupture (Lopes et al., 2025). In addition, increased sedimentation is known to reduce sea urchin grazing and promote *Sargassum* persistence (Kawamata et al., 2011). In contrast to leathery macroalgae, *Ulva* spp. declined to near absence during our monitoring and reappeared only in the summer of 2025. However, it remains unclear whether the dynamics of leathery and foliose macroalgae reflect a trajectory toward pre-disaster conditions, or are driven by other disturbances or stochastic processes.

Limited baselines are a significant impediment for assessing the full ecological consequences of the Fundão dam collapse and other major environmental impacts (Snowden, 1993; Underwood, 1997, 2000). This caveat ultimately elicits controversies about causal connections with ecological patterns reported in the aftermath of disasters, delaying effective action (Franco et al., 2024; Soares et al., 2020). It is also essential to recognize that disasters affect large areas that tend to be beyond the scope of monitoring programs targeted at the regular life-cycle of the industrial venture responsible for the event (Steinhauser et al. 2014; Girard & Fisher, 2018; Pinheiro et al., 2026), including its implementation, operation, and decommissioning. Also, such point-source monitoring programs most often do not include adequate baselines (i.e., >1 year before implementation) nor clear data-disclosure and review policies (e.g., Sánchez & Duarte, 2022).

In addition to the Fundão dam rupture, Brazil’s recent marine disasters include the 2019–2020 oil spill (Soares et al., 2020) and chemical spills in seaports (e.g., Rio Grande-1998, Rio de Janeiro-2000, Paranaguá−2004, Santos-1998 and 2015). However, adequate baselines were largely lacking, even in regions under critical risks (Martinez & Altvater, 2024; Martinez et al., 2022a). Environmental syntheses (e.g., Kowsmann, 2016), online databases (e.g., Petrobras, 2024), and Brazil’s Impact Reduction Plan (ICMBIO, 2020) represent important achievements of the recent decades, but are insufficient both for assessing disaster impacts (e.g., Soares et al., 2020) and for monitoring the chronic effects of industrialization (Sánchez & Duarte, 2022). Indeed, our spatial analyses revealed a countrywide mismatch between LTER sites and operations that may cause disasters, and this scenario tends to be rapidly aggravated under growing oil and gas and seaport infrastructure, as well as by the high-risk state of 20% of ~900 mining waste disposal dams (Freitas et al., 2022). The public-funded Brazilian LTER program aims to investigate ecosystem functioning, anthropogenic impacts and environmental changes (Tabarelli et al., 2013). However, its resources are often insufficient for implementing sampling designs that address multiple spatiotemporal scales (Tabarelli et al., 2013), especially in the coastal and marine realm. Only 10 LTER sites are currently operating in the country’s 3.6 million km^2^ Exclusive Economic Zone (EEZ), all in shallow waters. Distances of hundreds of kilometers between these operations and LTER sites can hinder impact detection, as the strongest effects near its source are unlikely to be captured. Moreover, responses at monitored sites far from the impact source may reflect natural and anthropogenic disturbances operating at different spatial and temporal scales, rather than the influence of the disaster. It is worth noting that the 2019 oil spill and the Fundão disaster affected over 3000 km and 500 km of coastline, respectively (Pinheiro et al., 2026; Soares et al., 2020).

The UN Office for Disaster Risk Reduction has recently called for a step-change in the mobilization of finance for adaptation and resilience (UNDRR, 2024). Although focused on natural hazards, the proposed mechanisms are useful as a starting point to rethink funding for baseline data collection and monitoring of disaster-prone marine areas, primarily from industries that are often responsible for marine disasters. While the annual budget for Brazilian marine LTER sites does not surpass USD 0.5 million (i.e., < USD 0.15 per km^2^ per year of the country’s EEZ), legal disputes over causal chains, effects, and the extent of marine disasters involve expenditures of tens of millions of USD, resulting in human health issues and hindering mitigation and restoration efforts (Johnston, 2012; Freitas et al., 2022). For instance, the results reported herein largely stem from “ex-post” impact assessments mandated by court rulings, as data from LTER and environmental licensing were unavailable. Finally, the Kunming-Montreal Global Biodiversity Framework (GBF), adopted in 2022 at COP15, sets global goals and targets to halt biodiversity loss by 2030, and also emphasizes the need for countries and businesses to take urgent action. Regular monitoring is a key activity to implement the GBF and provides opportunities to enhance accountability not only for impact assessment, but also for gauging biodiversity-positive impacts through ecosystem restoration, conservation, and sustainable practices.

## Conclusion

Our results show that spatiotemporal variability in coastal reef assemblages is structured across multiple scales, which limits post hoc attribution of environmental impacts when comprehensive baseline data are unavailable. This constraint is particularly acute in coastal and marine habitats that are heterogeneous in space and strongly seasonal, such as reefs and vegetated seabeds. Albeit our comparisons with pre-disaster data were limited to two sites with snapshot assessments carried out during the summer, they evidenced possible impacts from the disaster and signs of recovery after 7 years. Beyond this event, our mapping of industrial operations with potential to trigger marine disasters against the distribution of long-term ecological monitoring sites along the Brazilian coast reveals a marked spatial mismatch. As a result, disaster-related impacts are more likely to be missed, underestimated, or resolved only with high uncertainty. With high-risk coastal operations expanding globally, our findings support an urgent priority to establish representative, long-term coastal and marine observation networks that encompass regions exposed to anthropogenic disasters.

## Supplementary Information

Below is the link to the electronic supplementary material.

ESM1 (XLSX 10.5 KB)

## Data Availability

All data supporting the findings of this study are available within the paper, its Supplementary Information, and in a shiny application that is fully available online (https://abrolhos.shinyapps.io/coastal_assemblages/).
